# Associations between Polypharmacy, Self-Rated Health, and Depression in African American Older Adults; Mediators and Moderators

**DOI:** 10.3390/ijerph16091574

**Published:** 2019-05-06

**Authors:** Mohsen Bazargan, James Smith, Mohammed Saqib, Hamid Helmi, Shervin Assari

**Affiliations:** 1 Department of Family Medicine, Charles R Drew University of Medicine and Science, Los Angeles, CA 90095, USA; mobazarg@cdrewu.edu (M.B.); jamessmith@cdrewu.edu (J.S.); 2 Department of Family Medicine, University of California Los Angeles (UCLA), Los Angeles, CA 90095, USA; 3 Center for Research on Ethnicity, Culture, and Health, University of Michigan, Ann Arbor, MI 48109, USA; saqimoha@umich.edu; 4 Wayne State University, Detroit, MI 48202, USA; hhelmi@umich.edu

**Keywords:** African Americans, Black, older adults, polypharmacy, self-rated health, depression, depressive symptoms

## Abstract

*Background*. Despite the prevalence of multimorbidity among African American (AA) older adults, little information exists on correlates of polypharmacy (using 5+ medications) in AA older adults. There is more information available regarding the link between polypharmacy and physical aspects of health than subjective ones. *Aims*. In a local sample of AA older adults in Los Angeles, this study investigated the association of polypharmacy with self-rated health (SRH) and depression. We also explored gender differences in these links. *Methods*. This community-based study was conducted in south Los Angeles. A total number of 708 AA older adults (age ≥ 55 years) were entered into this study. From this number, 253 were AA men and 455 were AA women. Polypharmacy was the independent variable. Self-rated health (SRH) and depression were the dependent variables. Age, educational attainment, financial difficulty (difficulty paying bills, etc.), and marital status were covariates. Gender was the moderator. Multimorbidity, measured as the number of chronic diseases (CDs), was the mediator. Logistic regressions were applied for data analysis. *Results*. Polypharmacy was associated with worse SRH and depression. Multimorbidity fully mediated the association between polypharmacy and depressive symptoms. Multimorbidity only partially mediated the association between polypharmacy and poor SRH. Gender moderated the association between polypharmacy and SRH, as polypharmacy was associated with poor SRH in women but not men. Gender did not alter the association between polypharmacy and depression. *Conclusions*. AA older women with polypharmacy experience worse SRH and depression, an association which is partially due to the underlying multimorbidity. There is a need for preventing inappropriate polypharmacy in AA older adults, particularly when addressing poor SRH and depression in AA older women with multimorbidity.

## 1. Background

Polypharmacy, defined as the concomitant application of multiple medications [1,2] is positively associated with drug-drug interactions, drug side effects, and poor medication adherence [3,4]. Polypharmacy is an established risk factor for undesired and preventable [5,6,7] health outcomes [8] such as cognitive decline, falls, hospitalization, emergency visits, and mortality [3,4,9,10]. All of these conditions impose a considerable economic burden on the health care system and society [11]. At least some of these effects can be attributed to the association between polypharmacy and inappropriate use of medications (IUM) [12,13].

For multiple nested reasons, there is a need to study correlates of polypharmacy in older adults: First, population aging, which increases the relevance of studying polypharmacy [14]. This is because age is the largest contributing factor to the prevalence of polypharmacy, operating by increasing the development of chronic diseases and multimorbidity. Second, polypharmacy is closely associated with several health problems including morbidity and mortality [9,10]. Third, polypharmacy is more problematic in older adults than in younger adults, given their frailty and age-related physiological changes [15]. It has been mentioned that about 50% of older adults take at least one medication that is medically unnecessary [11], which increases the risk of adverse drug interactions and health care costs [11,16,17]. Fourth, inappropriate polypharmacy is responsible for 12% of all hospitalizations of older adults [18,19]. Fifth, a large proportion of such hospital admissions are preventable [20]. 

More is known about how polypharmacy is linked to multimorbidity, defined as having multiple chronic diseases (CDs) [21], than its relationship to self-rated health (SRH) and depression [22,23,24]. From the existing literature, very few studies have focused on African Americans (AAs) [2] and even fewer on AA older adults in particular [25]. While racial and ethnic groups differ in meaning and correlates of several health constructs [26], particularly SRH [27] and depression [28], there is a need to specifically study the links between polypharmacy, SRH, and depression in AA older adults. The results may help with health promotion within this community, particularly reducing inappropriate polypharmacy.

There is a need to study correlates of polypharmacy in AA older adults, and whether CD explains why individuals engaged in polypharmacy report depression and poor SRH. Polypharmacy often develops as a response to ageing [29,30], as older individuals are more likely to develop multiple CDs and their CDs require diagnosis and treatment [1,31]. As a result, polypharmacy is commonly a proxy of multimorbidity, and multimorbidity may be the underlying cause of individuals with polypharmacy rating their SRH as poor [30,32].

Unfortunately, there is a dearth of knowledge on epidemiological studies that investigate correlates of polypharmacy among AA older adults [2,25,33,34]. Although some research has been conducted on polypharmacy in this population [35,36,37], we know little about how elimination of polypharmacy would contribute to the elimination of racial disparities in health in the US. Any knowledge regarding correlates of polypharmacy in the AA older adults may help us with the design and implementations of interventions and programs that help AA older adults avoid potentially inappropriate medication use. 

Multiple factors may increase AA’s vulnerability to polypharmacy. Low health literacy, lower adherence, financial difficulties (difficulty paying bills, etc.), multiple competing health needs, and worse access to healthcare are all factors that may collectively increase AA’s risk of adverse events in the presence of polypharmacy [15]. We know that AA older adults have a lower chance of receiving the most effective medication regimens [38,39]. The lower quality care that AAs receive is due to biases in the healthcare system which place them at a higher risk of using simple older, generic medications with more complex dosing regimens [2,25]. Some of these disparities may contribute to a higher risk of inappropriate medication use or polypharmacy in AA older adults [40]. 

Gender is a central factor regarding risk and consequences of polypharmacy [41,42]. In several studies, women have shown a higher risk of polypharmacy [41,42,43]. Our previous study showed that in AA older adults, gender, comorbidity, potentially inappropriate medication use, as well as number of healthcare providers were associated with polypharmacy [25], which is itself associated with poor SRH and psychological distress [44]. However, gender alters determinants of SRH. That is, SRH seems to be shaped by more factors in women than in men [45], given the more inclusive nature of perception of health among women than men [45], also known as sponge hypothesis [45,46]. As a result, any studies on correlates of polypharmacy should test potential gender differences in these regards.

### Aims

This study aimed to investigate the associations between polypharmacy (taking 5+ medications) and SRH and depression among AA older adults. We tested whether multimorbidity (number of CDs) mediates this association and whether gender moderates it. 

## 2. Materials and Methods

### 2.1. Design and Setting

Data for this survey were collected from a cross-sectional study (survey) in south Los Angeles. The study was performed between 2015–2018 [47,48]. 

### 2.2. Institutional Review Board (IRB)

The study protocol was approved by the Institutional Review Board (IRB) of the Charles R. Drew University of Medicine and Science (CDU), Los Angeles (CDU IRB #: 14-12-2450-05). All participants signed a written informed consent before being enrolled to this study. Participants received financial compensation.

### 2.3. Process and Data Collection 

The data collection process included structured face-to-face interviews and a comprehensive assessment of medications. During the interviews, data on demographic factors (age and gender), SES (educational attainment, financial difficulty), multimorbidity (number of CDs), SRH, and depression were collected.

### 2.4. Participants

The study used a non-random sampling strategy to recruit AA older adults from areas in the south Los Angeles region such as the Watts area. Using a convenience sampling, AA older adults were eligible if they were AA/Black, were 55 years or older, and could complete an interview in English. Institutionalized participants were excluded from the study. Other exclusion criteria included being enrolled to any other clinical trials. Participants were sampled from 11 senior housing apartment units, 16 predominantly AA churches, and (low-income) public housing projects located in Service Planning Areas (SPAs) 6 in Los Angeles County. Church leaders and housing apartment managers facilitated and encouraged participation of the individuals in their communities. This sampling resulted in 740 AAs aged 55 years and older. The current analysis was limited to AA participants who were 55 years or older (*n* = 708).

All of our sampling was from residents of SPA 6 of the LA County. We selected SPA 6 due to 49% of older adults being AAs. Due to having a large land area (4300 square miles), LA County is divided into eight SPAs that allow the LA Department of Public Health to better conduct surveillance and provide public health services that are targeted to the specific needs of the populations. In SPA 6, 58% of adults have income levels less than 200% of the federal poverty line (FPL). Approximately 36% of adults in SPA6 are uninsured. From 2013–2015, the percentage of homeless AAs in SPA 6 has nearly doubled from 39% to 70% [47,48]. 

### 2.5. Measurements 

#### 2.5.1. Independent Variables

*Polypharmacy.* This study measured polypharmacy by a comprehensive evaluation of the medications. Polypharmacy in the current study was defined as taking 5+ medications [1]. 

#### 2.5.2. Dependent Variables

*Depression.* This study used the 15-item short Geriatric Depression Scale (GDS) to evaluate depression [26]. Responses were on a “yes” or “no” scale. A summary score was calculated with a potential range between 0–15. A higher score indicated more depression. The GDS-short form has excellent reliability and validity. This measure has been extensively used to measure depression among older adults in both clinical and community settings [49,50].

*Self-Rated Health.* We asked participants about their overall health. The responses ranged from excellent (1) to poor (5) [51]. We treated SRH as a continuous variable with a range from 1–5, where a higher score reflects worse health. Poor SRH predicts all-cause mortality in the general population [52,53] as well as patients with chronic disease [53]. Review articles and multiple original studies have established high predictive validity of poor SRH as a robust determinant of mortality risk, net of confounders such as SES and health [51].

#### 2.5.3. Mediator

*Multimorbidity.* Participants’ multimorbidity was operationalized as the number of CDs the participant had. Participants were asked about the presence of 11 CDs. Individuals were asked by the interviewer if a physician had ever told them that they have any of these following conditions: hypertension, heart disease, diabetes, lipid disorder/hypercholesterolemia, cancer, asthma, osteoarthritis, thyroid disorder, chronic obstructive pulmonary disease, rheumatoid arthritis, or gastrointestinal disease. Multimorbidity was a sum score that indicated number of CDs. Self-reports provide valid information regarding CDs [54,55,56], however, similar to any self-reported data, some bias (under-reporting or over-reporting) in this approach is expected. 

#### 2.5.4. Confounders

*Sociodemographic covariates.* Age, educational attainment, financial difficulty, and marital status were the sociodemographic variables in this study. Age was treated as a continuous variable. Educational attainment was operationalized as a continuous variable (years of schooling). Higher scores indicated more years of education. Self-reported (perceived) financial difficulty was measured using three items consistent with Pearlin’s list of main chronic financial difficulties experienced by low SES individuals [57,58,59]. The items asked the frequency by which a participant did not have enough money to afford clothing or food and had difficulty with paying bills. Responses ranged from 1 (never) to 5 (always). A sum score was calculated with a higher score reflecting more financial difficulty. Cronbach alpha (reliability measured by the average covariance between item-pairs, and the variance of the total score) of the measure in this study was 0.92.

#### 2.5.5. Moderator

Gender, our effect modifier, was treated as a dichotomous variable (1 women, 0 men).

### 2.6. Data Analysis

Data analysis was performed in SPSS 23.0. We used means, standard deviation (SD), and frequencies (%) to describe our variables in the pooled sample and also by gender. We used independent samples *t* test and Chi square to test the significant differences between AA men and AA women for all study variables. For multivariable analysis, we applied linear regression models. In our models, polypharmacy was the main independent variable, SRH or depression were the main outcome, and sociodemographic factors (age, educational attainment, financial difficulty, and marital status) were the covariates. Multimorbidity (number of CDs) was the mediator. Gender was the moderator. To test whether the association between polypharmacy and poor SRH/ depression is significantly larger for women than men, we used an interaction term between gender and polypharmacy. To test the mediation, we added the mediators to the model, after a basic model with the predictor. B (regression coefficient), standard errors (SE), t value, and p values were reported. 

## 3. Results

### 3.1. Descriptive Statistics

Table 1 describes the study variables in the pooled sample. This study included 708 AA older adults who were 55 years or older. Participants had an average age of 72 (SD = 8) years old. Most participants (about 64%) were AA women. Overall, about 72% had polypharmacy. 

Table 1 also compares the study variables between AA men and AA women. AA men were significantly younger than AA women in this study. AA women reported more financial difficulty than AA men. AA older men and women in this study did not differ in SRH or depression; however, AA women reported higher multimorbidity (number of CDs), compared to AA men. Polypharmacy was also more common in AA older women (77.4%) than AA older men (63.6%).

### 3.2. Bivariate Correlations

Table 2 shows the results of three correlation matrices one in the pooled sample, and then by gender. There was a positive correlation between polypharmacy and multimorbidity (number of CDs) in the pooled sample, AA men, and AA women. Polypharmacy was positively associated with age in the pooled sample and in AA women but not AA men. 

### 3.3. Multivariable Models

As Table 3 shows, polypharmacy was associated with worse SRH in the pooled sample, when multimorbidity was not controlled. Polypharmacy was still associated with SRH in the pooled sample, however, the effect of polypharmacy became smaller after controlling for multimorbidity, suggesting that multimorbidity partially mediates the association between polypharmacy and poor SRH. Gender significantly interacted with polypharmacy on poor SRH, suggesting that the association between polypharmacy and poor SRH is larger for AA women than AA men.

As Table 4 shows, polypharmacy was associated with higher depression in the pooled sample, when multimorbidity was not controlled. Polypharmacy was no more associated with depression in the pooled sample after multimorbidity was controlled, suggesting that multimorbidity fully mediates the association between polypharmacy and depression. Gender did not interact with polypharmacy on depression, suggesting that the association between polypharmacy and depression is not different for AA men and AA women.

## 4. Discussion

This study showed three main findings: First, polypharmacy was associated with poor SRH and depression in AA older adults, above and beyond confounders (age and SES). Second, the association between polypharmacy and poor SRH was moderated by gender, with poor SRH reflecting polypharmacy in AA women but not AA men. Third, multimorbidity (number of CDs) fully mediated the association between polypharmacy and depression. Similarly, multimorbidity partially mediated the association between polypharmacy and poor SRH. That is, it is incidence of CD, rather than polypharmacy, that probably correlates with depression in AA older adults. 

African American older adults who engage in polypharmacy also report worse SRH and higher depression. In a recent nationally representative study of AA adults older than 18 years old, polypharmacy was associated with higher mental distress in comparison to the individuals who did not use multiple medications. In that study, the association between polypharmacy and psychological distress was above and beyond multimorbidity [44]. That study, however, was not limited to older adults and included all adults. In addition, that study did not test whether multimorbidity is the underlying mechanism by which polypharmacy is associated with poor self-reported outcomes such as depression and SRH [44]. The current findings extend the result of previous studies by showing that multimorbidity may be why people who engage in polypharmacy are more depressed and report worse SRH.

In the current study, gender changed the association between SRH and polypharmacy. Poor SRH was indicative of higher odds of polypharmacy in AA women but not AA men. Gender differences in what SRH reflects is known. It is believed that SRH of women reflects a wider range of factors beyond multimorbidity [45,46], a phenomenon also called sponge hypothesis [45]. In this view, SRH is more inclusive in women than men [45]. For men, however, SRH only reflects multimorbidity. This is probably why poor SRH better reflects high risk of mortality for men than women [45]. 

Gender is a factor that influences both polypharmacy and SRH. Women are at an increased risk of polypharmacy [41,42]. At the same time, despite a lower risk of having fatal conditions, women report worse SRH than men [60]. Although there are also studies failing to show any gender differences in polypharmacy [61], several studies have documented a higher prevalence of polypharmacy in women than men [41,42,43]. Women are also more likely to receive potentially inappropriate medicine [14,42,43]. The effect of gender is seen in multiple ways. First, due to a gendered socialization [62], women are more likely to seek health care for their symptoms, and better communicate about their symptoms, so they are more likely to be diagnosed with multiple CDs [63]. In the presence of multiple CDs, women are more likely than men to seek professional care [64]. Due to the combination of being more aware of their health problems and symptoms [65] and more effective communication skills to describe their symptoms with health care providers, women tend to report worse SRH and more depression. Both CDs and depression are more likely to be diagnosed and treated in women than men [66]. At least for some men, seeking healthcare and disclosing poor health is seen as a sign of personal weakness [67,68,69]. Such beliefs may cause a delay in disclosing their symptoms to the health care providers [70]. This may result in a substantial delay in how and when men initiate healthcare utilization, compared to women [71]. Gender differences in health care use may result in some gender differences in medication prescription [72]. These differences may explain why polypharmacy, multimorbidity, depression, and poor SRH are more common in women than men. 

This study was limited to older adults. Age is a significant correlate of multimorbidity and polypharmacy. High age [42] is among the main determinates of polypharmacy and poor health, simply because aging is associated with a decline in normal function of body organs [31]. Similar to the literature on people in their sixties, our participants had a high prevalence of multimorbidity [73]. While CD is a risk factor, the real risk of polypharmacy on multi-morbidity is related to aging [74]. 

This was not a representative sample, but rather one that focused on residents of urban areas that struggle with poverty. This is very important due to the role of SES (education attainment, income, and employment) on polypharmacy [14,75,76]. At the same time, SES shapes subjective wellbeing [77]. Low SES indicators, such as financial problems, and low education negatively impact health and well-being [78]. In our data set, there was a negative association between financial problems and polypharmacy in the pooled sample and also in AA women. This association may suggest that AA older adults with considerable financial difficulty may be left out of the health care system, and their multimorbidity may not get the same prescriptions. At the same time, financial difficulty was associated with increased multimorbidity and poor SRH. While these are all important observations, they should not be generalized given the non-representative nature of our sample. We did not have data on income or occupation that may also have had a role in shaping population access to the health care system, which impacts polypharmacy. 

The associations between multimorbidity, polypharmacy, depression, and poor SRH should not be reduced to polypharmacy as a source of stress or distress. In individuals with polypharmacy, depression and poor SRH may be due to many factors such as more severe conditions, higher number of comorbid conditions, larger unmet health needs, and unmet psychological needs. Another explanation is that polypharmacy may generate distress itself. These hypotheses require additional research. 

### 4.1. Implications for Programs, Practice, and Research

Multimorbidity, polypharmacy, poor SRH, and depression are common and inter-related issues among AA older adults [79,80]. As these conditions cause morbidity and reduce quality of life [79,81,82], there is a need to address them jointly in health promotion programs within AA communities. Programs and interventions that are designed to promote health of AA older adults may simultaneously address multimorbidity, polypharmacy, depression, and SRH as common elements of poor health of AA older adults. In other terms, studies that address the health needs of the AA community should not discount or overlook polypharmacy, which is a close link to poor SRH and depression.

Future research should test whether prevention of inappropriate medication use, particularly inappropriate polypharmacy will enhance the subjective and physical health of AA older adults with multimorbidity [25,44]. There are not many evidence-based interventions that can be used effectively to reduce inappropriate polypharmacy among AA older adults [83,84]. As shown by the systematic reviews, the existing strategies are not very efficient in reducing inappropriate polypharmacy [85]. There is a need to evaluate the effect of interventions that screen AA older adults for unnecessary polypharmacy as well as associated morbidity [25,80]. Pharmacists may evaluate all medications and highlight the medications that are not safe or necessary, given a patient’s previous medical history, drug-drug interactions, or the Beers criteria [86]. Comprehensive programs and interventions may simultaneously assess individuals for multimorbidity, polypharmacy, poor SRH, and depression as comorbid problems, particularly in AA older women.

The results from this study may inform public health programs as well as clinical practice implications to promote the health of low-income AA communities. We argue that promotion of SRH and addressing depression in AA older adults should also address polypharmacy. That is, a comprehensive, wholistic approach that considers multimorbidity of AA older adults and accompanying polypharmacy as a part of health promotion programs should be used to promote SRH and address depression in AA older adults. Future research should test whether reducing inappropriate polypharmacy among AA older adults with multimorbidity would improve depression and enhance SRH. 

Among economically challenged AA older adults, gender may alter how polypharmacy due to multiple CDs impacts subjective well-being [87]. Addressing polypharmacy may have a more salient role in improving the subjective health of AA older women than AA older men. This again advocates for considering gender as a core element of health promotion of AA older adults. Gender-specific approaches may be needed to tackle the health disparities in the US. 

There is a need to conduct future comparative studies that can compare ethnic groups for the process that link multimorbidity, polypharmacy, depression, and SRH in AA and other ethnic groups. It is important to understand whether any of these links are more salient for AAs than other ethnic groups.

### 4.2. Limitations

Our study was not without limitations. The first limitation of our study was that because of the cross-sectional design of our study, we cannot make any causal inferences. Polypharmacy, depression, and poor SRH could all occur as a result of multimorbidity; however, a longitudinal study is needed to examine the causal links between these constructs. The direction of the associations of polypharmacy with objective vs subjective health measures may be different. Second, this study only measured the number rather than the type of medications. Without knowing the type of medications and quality of medications prescribed, it is impossible to know whether the participant is receiving inappropriate polypharmacy or not. Future work should also measure how individuals are receiving medications that increase the risk of harmful interactions. Third, we did not verify the self-reported CDs (multimorbidity) using other sources of information such as insurance data or medical charts. For example, we did not check refill information at the pharmacy level. Future research may collect CDs data using chart reviews. Fourth, some potential confounders were not measured. For example, we did not measure history of psychiatric problems and somatization problems. Our variables were all measured at an individual level, and providers data may also be relevant. Fifth, we did not collect data on disability and activities of daily living (ADLs), cognition, and frailty. Finally, the sample was a convenient sample of AA older adults in Los Angeles [24,32,33,46,47,87]. There is a need to replicate the findings reported here in other data sets such as Medicaid data. 

In particular, lack of information on the types and quality of participants’ medications, the considerable effect of likely drug-drug interactions, and the extent of patient medication adherence, contribute a high degree of uncertainty to the data analyzed here. Our study results are preliminary and may not directly improve health of AA populations. Despite these limitations, the results contribute to the current knowledge on how polypharmacy is associated with subjective well-being of AA older adults.

## 5. Conclusions

To summarize, among AA older adults, particularly for women, polypharmacy is associated with worse SRH and depression, and some of these associations are due to multimorbidity. Given the overlap between polypharmacy, poor SRH, multimorbidity, and depression, interventions that address inappropriate polypharmacy are needed in older adults who have poor physical and mental health. To address medication related challenges of AA older adults, knowing gender differences in how multimorbidity, SRH, depression, and polypharmacy are linked may be helpful. Such information may help with enhancing the efficacy of our interventions and ultimately improve the wellbeing and health of AA older population in underserved communities.

## Figures and Tables

**Table 1:** Descriptive characteristics in the pooled sample and by gender.

Characteristics	All	Men	Women
	*n* (%)	*n* (%)	*n* (%)
Gender			
Men	253 (35.7)	253 (100)	-
Women	455 (64.3)	-	455 (100)
Marital Status (Married) *			
No	612 (86.4)	205 (81.0)	407 (89.5)
Yes	96 (13.6)	48 (19.0)	48 (10.5)
Polypharmacy *			
No	195 (27.5)	92 (36.4)	103 (22.6)
Yes	513 (72.5)	161 (63.6)	352 (77.4)
	Mean (SD)	Mean (SD)	Mean (SD)
Age (Years) *	71.89 (8.22)	71.00 (8.15)	72.38 (8.23)
Educational Attainment	12.74 (2.24)	12.41 (2.51)	12.92 (2.06)
Financial Difficulty *	8.92 (5.46)	9.51 (6.13)	8.60 (5.03)
Multimorbidity (Number of CDs) *	3.83 (1.86)	3.52 (1.83)	4.00 (1.86)
SRH (1–5)	3.11 (1.01)	3.10 (1.08)	3.12 (0.97)
Depression	2.38 (2.74)	2.42 (2.68)	2.37 (2.77)

CDs: Chronic Diseases, SRH: Self-Rated Health. * *p* < 0.05.

**Table 2:** Bivariate associations between study variables overall and by gender.

	1	2	3	4	5	6	7	8	9
All									
1 Polypharmacy	1	0.33 **	0.06	0.02	0.13 **	−0.03	−0.10 **	0.03	0.15 **
2 Multimorbidity (Number of CDs)		1	0.27 **	0.31 **	−0.02	−0.08*	0.22 **	−0.02	0.12 **
3 SRH (Poor)			1	0.36 **	−0.20 **	−0.06	0.24 **	−0.09*	0.01
4 Depression				1	−0.23 **	−0.07	0.42 **	−0.06	−0.01
5 Age (Years)					1	−0.18 **	−0.29 **	−0.00	0.08 *
6 Educational Attainment						1	−0.09*	0.06	0.11 **
7 Financial Difficulty							1	−0.08 *	−0.08 *
8 Marital Status (Married)								1	−0.12 **
9 Gender (Women)									1
Men									
1 Polypharmacy	1	0.34 **	−0.03	−0.02	0.01	−0.01	−0.07	0.09	−
2 Multimorbidity (Number of CDs)			0.18 **	0.35 **	−0.05	−0.06	0.22 **	−0.04	−
3 SRH (Poor)				0.30 **	−0.26 **	−0.07	0.26 **	−0.13 *	−
4 Depression				1	−0.21 **	−0.05	0.39 **	−0.11	−
5 Age (Years)					1	−0.24 **	−0.31 **	0.02	−
6 Educational Attainment						1	−0.16 **	0.14 *	−
7 Financial Difficulty							1	−0.07	−
8 Marital Status (Married)								1	−
Women									
1 Polypharmacy	1	0.31 **	0.13 **	0.04	0.19 **	−0.07	−0.11 *	0.02	−
2 Multimorbidity (Number of CDs)		1	0.32 **	0.29 **	−0.02	−0.12**	0.23 **	0.03	−
3 SRH (Poor)			1	0.40**	−0.17 **	−0.05	0.23 **	−0.06	−
4 Depression				1	−0.24 **	−0.08	0.44 **	−0.04	−
5 Age (Years)					1	−0.15 **	−0.28 **	−0.00	−
6 Educational Attainment						1	−0.02	0.01	−
7 Financial Difficulty							1	−0.10 *	−
8 Marital Status (Married)								1	−

CDs: Chronic Diseases, SRH: Self-Rated Health. * *p* < 0.05. ** *p* < 0.001.

**Table 3:** Linear regressions on the associations between gender, polypharmacy, and self-rated health in the pooled sample.

	B	Std. Error	Beta	95% CI		*t*	Sig
Model 1							
Age (Years)	−0.02	0.00	−0.17	−0.03	−0.01	−4.45	0.000
Educational Attainment	−0.03	0.02	−0.07	−0.06	0.00	−1.74	0.082
Financial Difficulty	0.04	0.01	0.19	0.02	0.05	4.95	0.000
Married	−0.22	0.11	−0.07	−0.43	−0.00	−2.00	0.046
Gender (Women)	0.05	0.08	0.02	−0.10	0.21	0.66	0.511
Polypharmacy	0.23	0.08	0.10	0.07	0.40	2.81	0.005
Model 2							
Age (Years)	−0.02	0.00	−0.17	−0.03	−0.01	−4.44	0.000
Educational Attainment	−0.02	0.02	−0.04	−0.05	0.01	−1.12	0.264
Financial Difficulty	0.03	0.01	0.14	0.01	0.04	3.49	0.001
Married	0.00	0.08	0.00	−0.15	0.15	0.00	0.996
Gender (Women)	−0.24	0.11	−0.08	−0.44	−0.03	−2.23	0.473
Polypharmacy	0.06	0.09	0.03	−0.11	0.23	0.72	0.026
Multimorbidity (Number of CDs)	0.12	0.02	0.22	0.08	0.16	5.67	0.000
Model 3							
Age (Years)	−0.02	0.00	−0.18	−0.03	−0.01	−4.67	0.000
Educational Attainment	−0.02	0.02	−0.04	−0.05	0.01	−1.11	0.269
Financial Difficulty	0.03	0.01	0.14	0.01	0.04	3.51	0.000
Married	−0.23	0.11	−0.08	−0.43	−0.02	−2.13	0.033
Gender (Women)	−0.29	0.14	−0.14	−0.56	−0.02	−2.14	0.032
Polypharmacy	−0.18	0.13	−0.08	−0.43	0.07	−1.40	0.161
Multimorbidity (Number of CDs)	0.12	0.02	0.22	0.08	0.16	5.64	0.000
Polypharmacy × Gender (Women)	0.42	0.16	0.21	0.10	0.74	2.59	0.010

CDs: Chronic Diseases, SRH: Self-Rated Health. Model 1: Polypharmacy only. Model 2: Polypharmacy and Multimorbidity. Model 3: Model with the Interaction Term.

**Table 4:** Linear regressions on the associations between gender, polypharmacy, and depression in the pooled sample.

	B	Std. Error	Beta	95% CI		*t*	Sig
Model 1							
Age (Years)	−0.05	0.01	−0.14	−0.07	−0.02	−3.86	0.000
Educational Attainment	−0.08	0.04	−0.06	−0.16	0.01	−1.76	0.079
Financial Difficulty	0.19	0.02	0.38	0.15	0.22	10.45	0.000
Married	−0.26	0.27	−0.03	−0.80	0.28	−0.94	0.348
Gender (Women)	0.15	0.20	0.03	−0.24	0.54	0.74	0.460
Polypharmacy	0.43	0.21	0.07	0.01	0.85	2.03	0.043
Model 2							
Age (Years)	−0.05	0.01	−0.14	−0.07	−0.02	−3.88	0.000
Educational Attainment	−0.05	0.04	−0.04	−0.14	0.03	−1.22	0.221
Financial Difficulty	0.16	0.02	0.32	0.12	0.20	8.76	0.000
Married	−0.29	0.27	−0.04	−0.82	0.24	−1.09	0.277
Gender (Women)	−0.07	0.22	−0.01	−0.50	0.37	−0.30	0.762
Polypharmacy	0.00	0.20	0.00	−0.38	0.39	0.03	0.980
Multimorbidity (Number of CDs)	0.35	0.05	0.24	0.24	0.45	6.48	0.000
Model 3							
Age	−0.05	0.01	−0.14	−0.07	−0.02	−3.99	0.000
Educational Attainment	−0.05	0.04	−0.04	−0.14	0.03	−1.22	0.224
Financial Difficulty	0.16	0.02	0.32	0.12	0.20	8.76	0.000
Married	−0.28	0.27	−0.03	−0.81	0.25	−1.03	0.302
Gender (Women)	−0.38	0.35	−0.07	−1.06	0.30	−1.08	0.279
Polypharmacy	−0.38	0.32	−0.06	−1.01	0.25	−1.18	0.239
Multimorbidity (Number of CDs)	0.35	0.05	0.24	0.24	0.45	6.45	0.000
Polypharmacy × Gender (Women)	0.55	0.42	0.10	−0.26	1.37	1.33	0.184

CDs: Chronic Diseases, SRH: Self-Rated Health. Model 1: Polypharmacy only. Model 2: Polypharmacy and Multimorbidity. Model 3: Model with the Interaction Term.
